# Biological characterization and antibacterial effects of a novel virulent *Acinetobacter baumannii* phage strain vB_Aba_QH4 *in vitro* and *in vivo*

**DOI:** 10.3389/fmicb.2025.1638702

**Published:** 2025-09-26

**Authors:** Li Ruizhe, Cheng Peng, Li Zian, Zhou Jianwu, A. Xiangren

**Affiliations:** ^1^Basic Medicine Department of Medical College, Qinghai University, Xining, China; ^2^Department of Clinical Laboratory, Qinghai Provincial People's Hospital, Xining, China

**Keywords:** *Acinetobacter baumannii*, phage, biological characterization, genomic analysis, phage therapy

## Abstract

**Background:**

*Acinetobacter baumannii* is a major opportunistic pathogen in hospital-acquired infections, posing challenges for clinical control. This study aimed to isolate and characterize a novel strain of *A. baumannii* phage from hospital wastewater and to determine its biological properties and evaluate its *in vitro* and *in vivo* bacteriostatic effects.

**Methods:**

Using *A. baumannii* NO.424 as the host bacterium, phages were isolated and purified using the plaques method and double-layer agar method. The host spectrum and phage microscopic morphology were determined using the plaques method and transmission electron microscopy, respectively. The Optimal multiplicity of infection, adsorption curve, one-step growth curve, thermal stability, acid–base stability, UV tolerance, and chloroform stability were determined using the double-layer agar method. Bioinformatic functional analysis was performed after genome sequencing using the Illumina platform. The *in vitro* and *in vivo* inhibitory effects were determined using the turbidimetric method and wax borer larvae animal model, respectively.

**Results:**

A lytic phage vB_Aba_QH4 with a narrow host spectrum was successfully isolated and purified from a hospital wastewater sample using *A. baumannii* NO.424 as the host bacterium. vB_Aba_QH4 microscopic morphology head is positively icosahedral (60 ± 2 nm) with a retractable tail (107 ± 2 nm). vB_Aba_QH4 has an optimal MOI of 0.001, an adsorption rate of 99.99% at 6 min, an incubation period of 10 min, a cleavage period of 40 min, and a burst size of 460.5 PFU/cell. The bioactivity of vB_Aba_QH4 remained stable at 4 °C to 50 °C, and pH of 3–11. vB_Aba_QH4 was inactivated by UV light after 40 min of irradiation, and chloroform solution had no significant effect on the bioactivity of vB_Aba_QH4. Furthermore, vB_Aba_QH4 has a linear double-stranded DNA with a length of 45,017 bp and a GC content of 37.25%, with a total of 89 predicted open reading frames. vB_Aba_QH4 showed significant inhibition of *A. baumannii* within 12 h, with an inhibition rate exceeds 90%. The highest survival rates were 95 and 70% in the treatment and prevention groups, respectively.

**Conclusion:**

vB_Aba_QH4 possesses strong environmental tolerance, rapid adsorption capacity, and good cleavage power, with *in vitro* and *in vivo* bacterial inhibition experiments showing good antimicrobial effects.

## 1 Introduction

*Acinetobacter baumannii* is one of the most challenging hospital-acquired pathogens that expresses a variety of virulence factors leading to its high pathogenicity (Rodman et al., 2019). The bacterium mainly infects critically ill patients and can cause multisystem clinical conditions, including respiratory pneumonia, bloodstream infections, and central nervous system infections (Maragakis and Perl, 2008).

Current clinical anti-infective treatment for *A. baumannii* mainly relies on antibiotics. However, antibiotic treatment has various adverse effects, including dermatologic adverse effects, irritation of the mucosa of the digestive system, and drug-induced liver injury (Björnsson, 2017; Zhuo et al., 2022). More importantly, the extensive drug resistance and high fatality rate associated with *A. baumannii* present a formidable challenge to clinical anti-infective therapies (Ibrahim et al., 2021). At present, the pace of research and development for new antibiotics has slowed considerably (Hatfull et al., 2021). Against this backdrop, phage therapy has demonstrated significant potential as an alternative therapeutic strategy within the realm of antimicrobial interventions (Kortright et al., 2019). Phages are a class of viruses that naturally lyse specific bacteria (Liu B. et al., 2022). They are widely distributed in nature, with a total abundance of up to 1,031, mainly in the human microcosm, soil, and water (Doi et al., 2015; Kang et al., 2022). Phages are strictly host-specific, enabling precise targeted removal of pathogenic bacteria. The targeted treatment mode can effectively kill the target strain, avoiding the destruction of normal flora and reducing adverse reactions. Furthermore, phages can also lyse drug-resistant bacteria (Aslam et al., 2019).

Therefore, phages are increasingly recognized as promising antimicrobial agents and have gained renewed attention from researchers in recent years (Totten et al., 2022). Screening of novel virulent phages is important for antimicrobial therapy. During phage-bacteria interactions, several key factors determine their therapeutic efficacy, including host spectrum, adsorption capacity, lysis properties, genome-wide characterization, antimicrobial activity, and temperature and pH tolerance range. This study aimed to determine the biological properties and *in vitro* and *in vivo* inhibitory effects of a novel virulent *A. baumannii* phage vB_Aba_QH4.

## 2 Materials and methods

### 2.1 Strains and growth conditions

All strains used in this study were obtained from clinical isolates stored at Qinghai Provincial People’s Hospital. Strain identification was performed using a Matrix-Assisted Laser Desorption/Ionization Time-of-Flight Mass Spectrometry (MALDI-TOF MS, microflex Bruker, Germany), and the strains were preserved at −80 °C in LB medium containing 30% glycerol (Donghuan Kai Microbial Technology Co., Ltd.).

*Acinetobacter baumannii* NO.424 was isolated in June 2024 from a sputum specimen of a patient with pulmonary contusion. All phage biological characteristic measurements and *in vitro* and *in vivo* antimicrobial activity experiments in this study used *A. baumannii* NO.424 as the experimental strain. *A. baumannii* NO.424 was identified using MALDI-TOF MS, and multi-locus sequence typing (MLST) was performed using the method described by Kaiser (Kaiser et al., 2009). After analysing the seven housekeeping genes of the strain using the MLST 2.0 online platform, the MLST of *A. baumannii* NO.424 was determined to be type 2411 (Supplementary Table 1).

The growth curve of *A. baumannii* NO.424 was determined using spectrophotometry. When the OD600 value was between 0.2 and 0.9, *A. baumannii* NO.424 was in the logarithmic growth phase (Supplementary Figure 1). When the OD600 value is 0.75–0.8, the number of *A. baumannii* NO.424 bacteria is 1 × 108 CFU/mL. Take bacterial culture with an OD600 of 0.75–0.8, perform a 10-fold serial dilution with physiological saline, then pipette 10 μL of the appropriately diluted bacterial culture onto LB agar plates. Incubate at 37 °C for 18–24 h, then count colonies within the range of 30–300 CFU. The original bacterial suspension concentration is calculated using the following formula:

$$\begin{array}{l} Bacterialconcentration\;\left(CFU/mL\right)=Averagecolonycount\\ \;\;\;\;\;\;\;\;\;\;\;\;\;\;\;\;\;\;\;\;\;\;\;\;\;\;\;\;\;\;\;\;\;\;\;\;\;\;\;\;\;\;\;\;\;\;\;\;\;\;\;\;\;\;\;\;\;\;\;\;\;\;\;\;\;\;\;\;\;\;\;\;\;\;\;\;\;\;\;\;\;\;\;\;\;\times Dilutionfactor\times 10 \end{array}$$

### 2.2 Phage enrichment, isolation, and purification

Phage enrichment, isolation, and purification from hospital wastewater were performed according to the method described by Sattar along with some modifications (Sattar et al., 2022). During enrichment, a phage filtrate was obtained by centrifuging the effluent samples at 10,000 × *g* for 5 min and then filtering it using a 0.22-μm microporous filter membrane (Millipore, USA). Afterwards, 10 mL of the filtrate, 10 mL of 2 × LB broth, and 200 μL of logarithmic growth phase NO.424 bacterial suspension were mixed. The mixture was then incubated for 12–16 h at 37 °C and 180 rpm with shaking before centrifugation, filtration, and collection of the enriched solution.

Phage isolation was performed using the plaques method: 100 μL of logarithmic growth phase NO.424 bacterial suspension was mixed with 5 mL of semi-solid medium (0.6% agar, 50 °C) and poured onto LB solid agar plates (1.5% agar) measuring 9 × 9 cm. After the medium solidified, 10 μL of the enriched solution was applied to the plates and incubated at 37 °C for 18 h to observe the generation of plaques.

Purification was performed using the double-layer agar method: individual plaques were picked and diluted to the appropriate ratio using gradient multiplicity dilution in phosphate-buffered saline (PBS) to obtain virus dilutions. Afterwards, 100 μL of virus dilution was added to an equal volume of logarithmic growth phase NO.424 bacterial suspension before adding 5 mL of 50 °C semi-solid medium, which was then poured onto an LB solid plate. The medium was allowed to solidify and then incubated at 37 °C for 18 h before being examined for the formation of plaques. The purification process was repeated at least three times until the plaques morphology was homogeneous.

### 2.3 Phage host range determination

The phage host range was determined using the plaque assay and double-layer agar plate method (Huang et al., 2023), covering 80 strains of *A. baumannii* and 20 strains of *Pseudomonas aeruginosa*. MLST analysis was performed on the lysate-positive strains.

### 2.4 Phage morphology by transmission electron microscopy

Amplify the phage using the double agar method. Once numerous plaques have formed on the plate, add 10 mL of LB liquid medium and incubate at 37 °C and 180 rpm for 4 h to elute the phage. The eluate is transferred to a centrifuge tube and centrifuged at 10,000 × *g* for 5 min to remove impurities. The supernatant is then filtered through a 0.22 μm filter membrane to obtain a purified phage suspension. The titer of this suspension must be ≥ 10^10^ PFU/mL to be used in subsequent experiments (Cheng et al., 2025).

Using a pipette, 20 μL of the phage suspension was dispensed onto a copper grid and allowed to adsorb naturally for 5–10 min. Excess droplets were removed using filter paper strips, and the sample was left to dry slightly. Then, 20 μL of 2% phosphotungstic acid solution was dispensed onto the copper grid using a pipette and left to stand for 3–5 min. Excess liquid was again removed with filter paper, and the grid was dried under white light. Observation and imaging were performed using a transmission electron microscope (TEM, Hitachi HT7700, Japan) at an acceleration voltage of 80 kV (Xue et al., 2020). The head width and tail length of viral particles were measured using Image-Pro Plus 6.0 software.

### 2.5 Optimal multiplicity of infection

The experimental method described by Peters (Peters et al., 2023) was used, with some modifications, to determine the optimal multiplicity of infection (MOI). Approximately 1 mL of phage suspension (10^10^ –10^4^ PFU/mL) and an equal volume of logarithmic growth phase NO.424 bacterial suspension (10^8^ CFU/mL) were mixed to achieve MOIs of 100, 10, 1, 0.1, 0.001, and 0.0001, respectively. The mixture was incubated at 37 °C and 180 rpm with shaking for 12–16 h. The lysate was filtered using a 0.22-μm filter to collect the filtrate and determine the phage titer using the double-layer agar method.

$$\begin{array}{l} Phagetitre\;\left(PFU/mL\right)=Meannumberofphageplaque\\ \;\;\;\;\;\;\;\;\;\;\;\;\;\;\;\;\;\;\;\;\;\;\;\;\;\;\;\;\;\;\;\;\;\;\;\;\;\;\;\;\;\;\;\;\;\;\;\;\;\;\;\;\;\;\times Dilution\times 10 \end{array}$$

The MOI with the highest phage titer was the best MOI (Li et al., 2019). The process was repeated three times independently.

### 2.6 Adsorption rate

Phage adsorption rate is an important factor in determining the efficiency of infection (Yang et al., 2010). After mixing 10 mL of phage suspension (10^5^ PFU/mL) with an equal volume of logarithmic growth phase NO.424 bacterial suspension (10^8^ CFU/mL) according to the optimal MOI, the mixture was incubated for 20 min at 37 °C and 180 rpm with shaking, during which samples were taken every 2 min, and the lysate was filtered through a 0.22-μm filter membrane to collect the filtrate and determine the phage titer.

$$Adsorptionrate\;\left(\%\right)=\left[\begin{array}{l} \left(\begin{array}{l} Initialphagetiter-\\ Phagetiterinfiltrate \end{array}\right)\\ /Initialphagetiter \end{array}\right]\;\times \;100\%.$$

The process was repeated three times independently.

### 2.7 One-step growth curve

A one-step growth curve assay was performed to assess phage latency and burst (Tan et al., 2021). After mixing 1 mL of phage suspension (10^5^ PFU/ml) with an equal volume of logarithmic growth phase NO.424 bacterial suspension (10^8^ CFU/mL) according to the optimal MOI, the mixture was incubated at 37 °C and 180 rpm for 10 min. The mixture was centrifuged at 10,000 × *g* for 5 min at 4 °C and the supernatant was discarded. The precipitate was washed with PBS, and the procedure was repeated twice. Finally, the precipitate was resuspended in 10 mL of LB broth and incubated for 1 h at 37 °C and 180 rpm, during which samples were taken every 10 min to determine the phage titer. PFU in the early incubation period (before lysis) represents the number of bacteria initially infected *(N0)*. PFU after lysis represents the total number of daughter phages released *(Nt)*. And calculate the burst size of vB_Aba_QH4 according to the following formula:

Burstsize(PFU/cell)=(Nt−N0)/N0

The experiment was repeated three times.

### 2.8 Physical and chemical stability

The stability of the phage under physicochemical factors was determined according to a previously described method with slight modifications (Evseev et al., 2021). Regarding temperature stability, 1 mL of 10^8^ PFU/mL phage suspension was added to a centrifuge tube, and the phage titer was determined after 1 h in a water bath at 4 °C, 25 °C, 37 °C, 50 °C, 60 °C, 70 °C, and 80 °C, respectively. For pH stability, 100 μL of 10^8^ PFU/mL phage suspension was mixed with 900 μL of different pH buffers (1–12), and the phage titer was determined after 1 h in a 37 °C water bath. The phage titer was determined after being placed for 1 h in a 37 °C water bath. For UV radiation stability, 10 mL of 10^8^ PFU/mL phage suspension was taken in a petri dish and irradiated by UV light (20 W, *λ* = 254 nm, distance 30 cm) for 1 h in a biosafety cabinet, and samples were taken every 10 min to determine the phage titer. During chloroform assay, 10^8^ PFU/mL phage suspension was mixed with chloroform so that the final chloroform concentration of the mixture was 0, 1, 2, 3, 4, and 5%, respectively. The phage titer was measured after 1 h in a water bath at 37 °C to determine whether the phage strain contained lipids (He et al., 2024). Each of the above experiments was repeated three times.

### 2.9 Phage genome sequencing and bioinformatics analysis

DNA was extracted using the QIAamp DNA Mini Kit (Qiagen, Hilden, Germany), and the complete genome was sequenced using Illumina’s MiSeq sequencing platform (Thermo Fisher Scientific, USA) and PromethION sequencer (Oxford Nanopore Technologies, Oxford, UK) for complete genome sequencing (Liu S. et al., 2022). Quality control and low-quality sequences were performed using fastp (version: 0.23.2) (Chen et al., 2018; Fan et al., 2018). Sequences were assembled using Unicycler (version: 0.5.0) (Valappil et al., 2021). Open reading frame (ORF) prediction analysis was performed using Prodigal (version: v2.6.3) (Rungsirivanich et al., 2021). Gene circle maps were drawn via the online tool Proksee (Yadav et al., 2022). Per. Ident analysis was performed using the NCBI BLAST algorithm (Lu et al., 2017). MEGA7 software (neighborhood method, bootstrap value set to 1,000) was used to construct a complete genome phylogenetic tree and a terminase large subunit protein phylogenetic tree (Lin et al., 2016). The PhageAI online platform was used to evaluate the integrity of phage genomes and predict the lifestyle of phage (Marongiu et al., 2021). Antimicrobial resistance genes (AMRs) and virulence genes were screened in the Comprehensive Antibiotic Resistance Database (Alcock et al., 2022) and VirulenceFinder (Roer et al., 2024).

### 2.10 *In vitro* bacterial inhibition assay

The inhibitory effect on bacterial growth was determined by measuring the OD600 absorbance value of phage-bacteria mixed cultures (Moreira et al., 2016). In the experimental group, 100 μL of phage suspension (10^10^–10^4^ PFU/mL) and an equal volume of logarithmic growth phase NO.424 bacterial suspension (10^8^ CFU/mL) were added into 96-well plates so that the MOIs were 100, 10, 1, 0.1, 0.01, 0.001, and 0.0001, respectively. In the control group, 100 μL of LB broth medium and an equal volume of NO.424 bacterial suspension (10^8^ CFU/mL) were mixed. The 96-well plates were incubated at 37 °C and 180 rpm with shaking for 12 h, during which the OD600 absorbance value was measured using a microplate reader (Sunrise, Tecan, Switzerland) at 30 min intervals. The experiments were performed in triplicate and independently repeated three times.

### 2.11 *In vivo* bacterial inhibition experiments

Host bacterial lethal concentrations were assessed using an infection model of large wax borer larvae (Olszak et al., 2015) (*Galleria mellonella* purchased from Huiyuide Biotechnology Company, Tianjin, China). The larvae (body length 30 ± 5 mm, body weight 300 ± 15 mg) were selected and randomly divided into seven groups (*n* = 10/group) after disinfecting the abdominal epidermis with 75% ethanol and were injected with 10 μL of PBS (blank control), 10 μL of phage suspension at 10^9^ PFU/ml and 10 μL of suspension of NO.424 in the logarithmic growth phase (10^4^–10^8^ CFU/mL) in a petri dish lined with sterilized filter paper and incubated for 72 h at 37 °C, 60% humidity, and protected from light.

The larvae were randomly divided into 15 groups (*n* = 20/group) after disinfecting the abdominal epidermis with 75% ethanol. The infection group was injected with 10 μL of 10^7^ CFU/mL NO.424 bacterial suspension for 1 h, followed by 10 μL of PBS. The treatment group was injected with 10 μL of 10^7^ CFU/mL NO.424 bacterial suspension for 1 h followed by 10 μL of phage suspensions of different titers (10^3^–10^9^ PFU/mL). The prophylactic group was injected in the reverse order of the treatment group. After the injections, the larvae were kept at 37 °C and 60% humidity while avoiding direct light while assessing their survival. The survival rates were recorded every 8 h up to 72 h. The criterion for death determination was the absence of motor response to mechanical stimulation (Insua et al., 2013).

### 2.12 Statistical analysis

All data were analyzed using GraphPad Prism 10.1.2 software and are expressed as means and standard deviations. Among the physicochemical factors stability and *in vitro* bacterial inhibition experiments data were analyzed using two-way analysis of variance (ANOVA). Survival curves were analyzed using the log-rank (Mantel-Cox) test. Furthermore, *p*-values < 0.05 were considered statistically significant.

## 3 Results

### 3.1 Morphological characteristics

**Figure 1:**
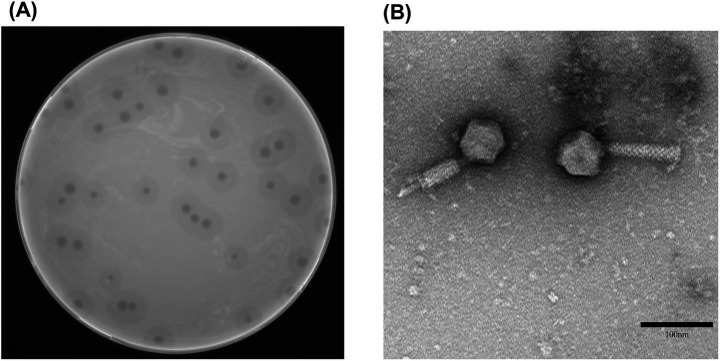
Morphological characteristics of phage vB_Aba_QH4. **(A)** Morphology of phage plaques on double-layer agar medium. **(B)** Morphology of phage vB_Aba_QH4 observed by transmission electron microscopy. Scale bar = 100 nm.

vB_Aba_QH4 presented a uniform and transparent plaque with a diameter of 2–3 mm on the double-layer AGAR medium, accompanied by obvious halo phenomena; the diameter of the halo ring was up to 7–8 mm (Figure 1A). This indicates that vB_Aba_QH4 is a lytic phage. Under the electron microscope, the head of vB_Aba_QH4 (60 ± 2 nm) is a regular icosahedral structure, and its tail measures 107 ± 2 nm, making a scalable myotail structure belonging to a typical Myoviridae (Figure 1B).

### 3.2 Host spectrum

Among the 100 strains selected for this experiment, vB_Aba_QH4 was only able to lyse two strains of *A. baumannii*, Except for the experimental strain *A. baumannii* NO.424, only the *A. baumannii* NO.307 strain can be lysed. The MLST of *A. baumannii* NO.307 was determined using the same method, and the result showed it was also a 2411 type (Supplementary Table 2). The remaining 78 *A. baumannii* strains and 20 *Pseudomonas aeruginosa* strains were not lysed by vB_Aba_QH4 and showed negative results.

### 3.3 MOI, adsorption, and one-step growth curve analysis

**Figure 2:**
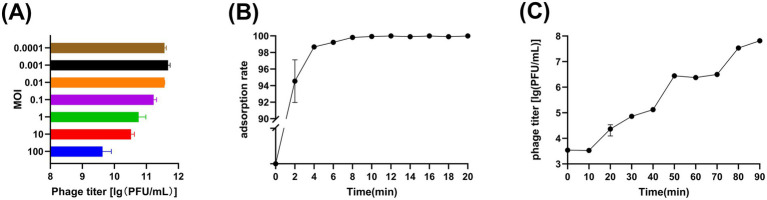
Growth characteristics of phage vB_Aba_QH4. **(A)** Phage vB_Aba_QH4 titers under different MOIs (phage-to-bacteria ratio = 0.0001, 0.001, 0.01, 0.1, 1, 10, 100) are depicted on the *x*-axis. **(B)** Adsorption rate curve of phage vB_Aba_QH4 (MOI = 0.001). **(C)** One-step growth curve of phage vB_Aba_QH4 (MOI = 0.001).

The phage titres at different MOIs were as follows: 4.27 × 10^9^ PFU/mL at MOI = 100, 3.32 × 10^10^ PFU/mL at MOI = 10, 5.75 × 10^10^ PFU/mL at MOI = 1, 1.69 × 10^11^ PFU/mL at MOI = 0.1, 3.71 × 10^11^ PFU/mL at MOI = 0.01, 4.75 × 10^11^ PFU/mL at MOI = 0.001 and 3.66 × 10^11^ PFU/mL PFU/mL at MOI = 0.0001. It is obvious that when MOI = 0.001, the peak value of vB_Aba_QH4 was the highest, with a titer of 4.75 × 10^11^ PFU/mL, indicating that the optimal MOI was 0.001 (Figure 2A). Furthermore, The adsorption rate of vB_Aba_QH4 reached 94.53% at 2 min, 98.67% at 4 min, and 99.99% at 6 min (Figure 2B). The one-step growth curve indicates that the incubation period of vB_Aba_QH4 phage was approximately 10 min, the lysis period was approximately 40 min, and the burst size was 460.5 PFU/cell (Figure 2C).

### 3.4 Stability of physical and chemical factors

**Figure 3:**
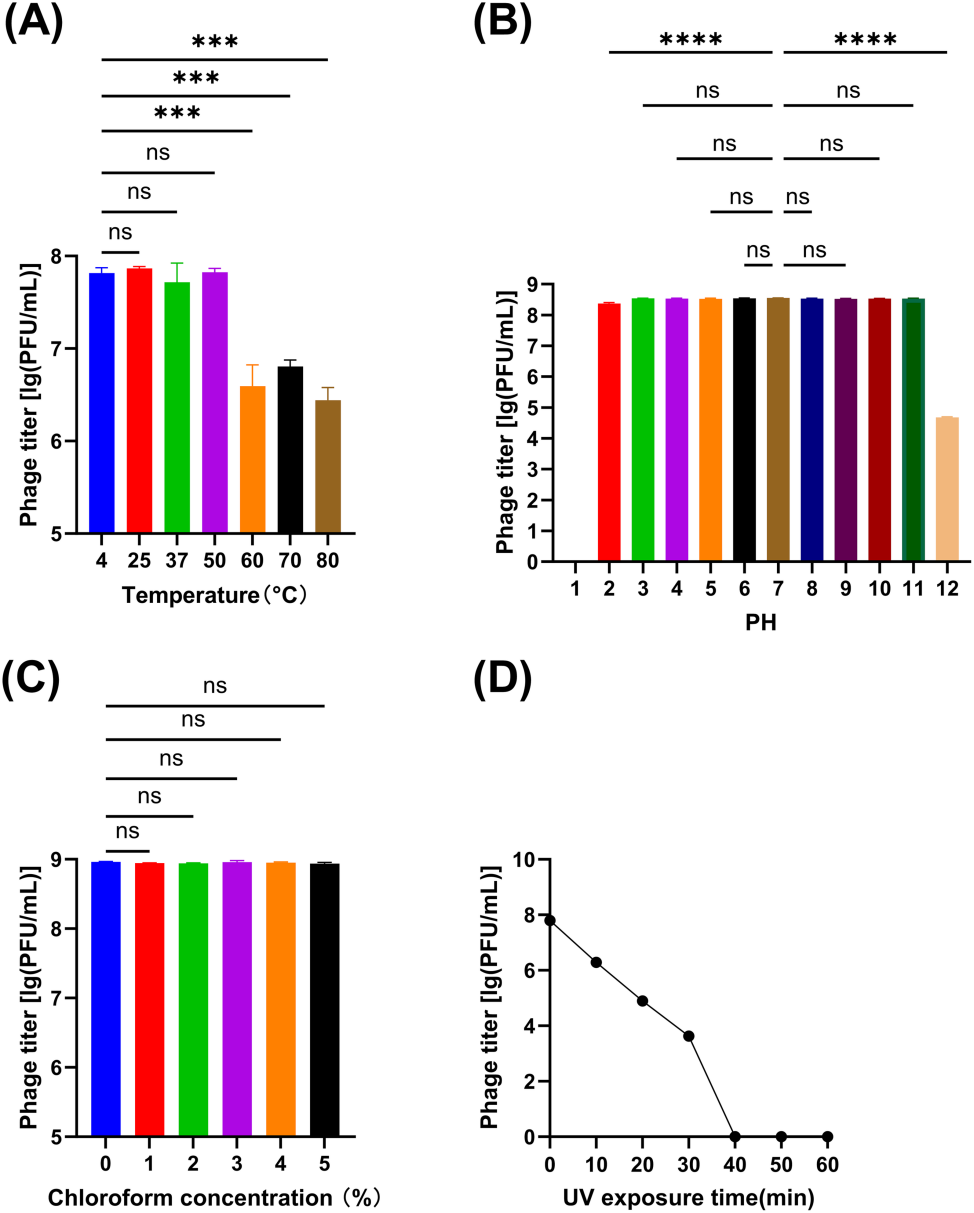
Physiochemical stability of phage vB_Aba_QH4. **(A)** Stability of vB_Aba_QH4 at different temperatures. **(B)** Stability of vB_Aba_QH4 at different pH values. **(C)** Stability of vB_Aba_QH4 at different chloroform concentrations. **(D)** Stability of vB_Aba_QH4 under UV radiation with different irradiation durations. **(A–C)**: Statistical analyses were performed using one-way ANOVA. Significance levels are indicated as follows: ns for non-significance; *p < 0.05; **p < 0.01; ***p < 0.001; ****p < 0.0001.

The titer changes of phage vB_Aba_QH4 within the range of 4 °C to 50 °C were not statistically significant (*p* > 0.05); however, a significant decrease in titer was observed between 60 °C to 80 °C (*p* < 0.001) (Figure 3A). Within the pH range of 3–11, there was no statistically significant change in the titer of phage vB_Aba_QH4 (*p* > 0.05). The titer significantly decreased at pH values of 2 and 12 (*p* < 0.0001); when the pH value was 12, the titer decreased from 10^8^ PFU/mL to 10^5^ PFU/mL and completely lost its activity at pH = 1 (Figure 3B). Within the chloroform concentration range of 0 to 5%, the change in the titer of phage vB_Aba_QH4 was not statistically significant (Figure 3C). The activity of phage vB_Aba_QH4 gradually decreases after exposure to UV light, until it completely loses its activity after 40 min (Figure 3D).

### 3.5 Genomic analysis and functional annotation

The vB_Aba_QH4 (NCBI serial number: PQ227708) gene is a linear double-stranded DNA, with a total gene length of 45,107 bp and a CG content of 37.25%. Using Prodigal, 89 open reading frames (ORFs) (Supplementary Table 3) were predicted, among which 77 were in the positive chain and 12 were in the negative chain. The results of functional annotation indicated that 26 ORFs were successfully endowed with biological functions and mainly participated in key life cycle processes such as viral replication and transcription, structural packaging, and host lysis. A total of seven replication and transcription-related proteins were predicted. They are, respectively, ORF7 (DNA N-6-adenine-methyltransferase), ORF10 (putative replicative DNA helicase), ORF21 (nucleotide kinase), ORF85 (DNA exonuclease), ORF86 (Erf-like ssDNA annealing protein), ORF2 (transcriptional regulator), and ORF31 (F-box domain protein); a total of 12 structure-related proteins were predicted, namely ORF34 (AB1gp35), ORF49 (minor head protein), ORF50 (major head protein), ORF57 (head-tail adaptor), ORF60 (DUF3383 family protein), ORF65 (tail tube initiator-like protein), ORF66 (virion structural protein), ORF69 (baseplate hub), ORF72 (tail sheath initiator protein), ORF73 (baseplate J-like protein), ORF74 (structural protein) and ORF77 (AB1gp75); Four package-related proteins were predicted, namely, ORF37 (terminase large subunit), ORF38 (portal protein), ORF48 (head maturation protease) and ORF58 (cysteine protease). Additionally, three proteins were related to lysozyme, namely ORF16 (immunity to superinfection), ORF64 (Lysozyme-like protein), and ORF79 (lysozyme). The remaining 72 ORFs are tentatively designated as hypothetical proteins, among which four are predicted to be presumed proteins: ORF36 (putative terminase small subunit), ORF70 (putative baseplate assembly protein), ORF75 and ORF76 (putative tail fiber protein).

**Figure 4:**
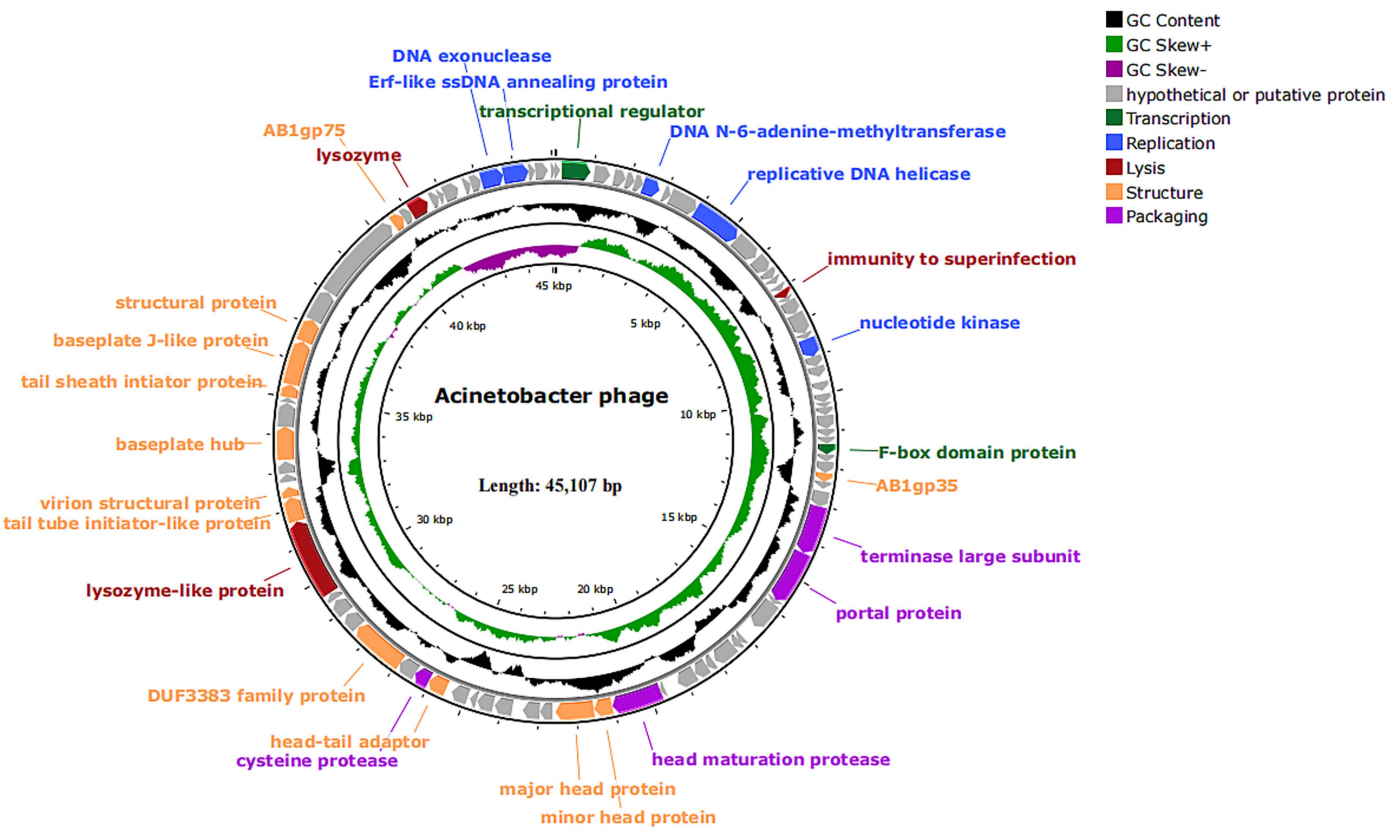
Genome map of phage vB_Aba_QH4. All predicted ORFs are indicated by arrows. Colours denote functional categories: black, content; light green, GC skew+; dark purple, GC skew-; grey, hypothetical or putative protein; dark green, transcription; blue, replication; red, lysis; orange, structure; light purple, packaging.

**Figure 5:**
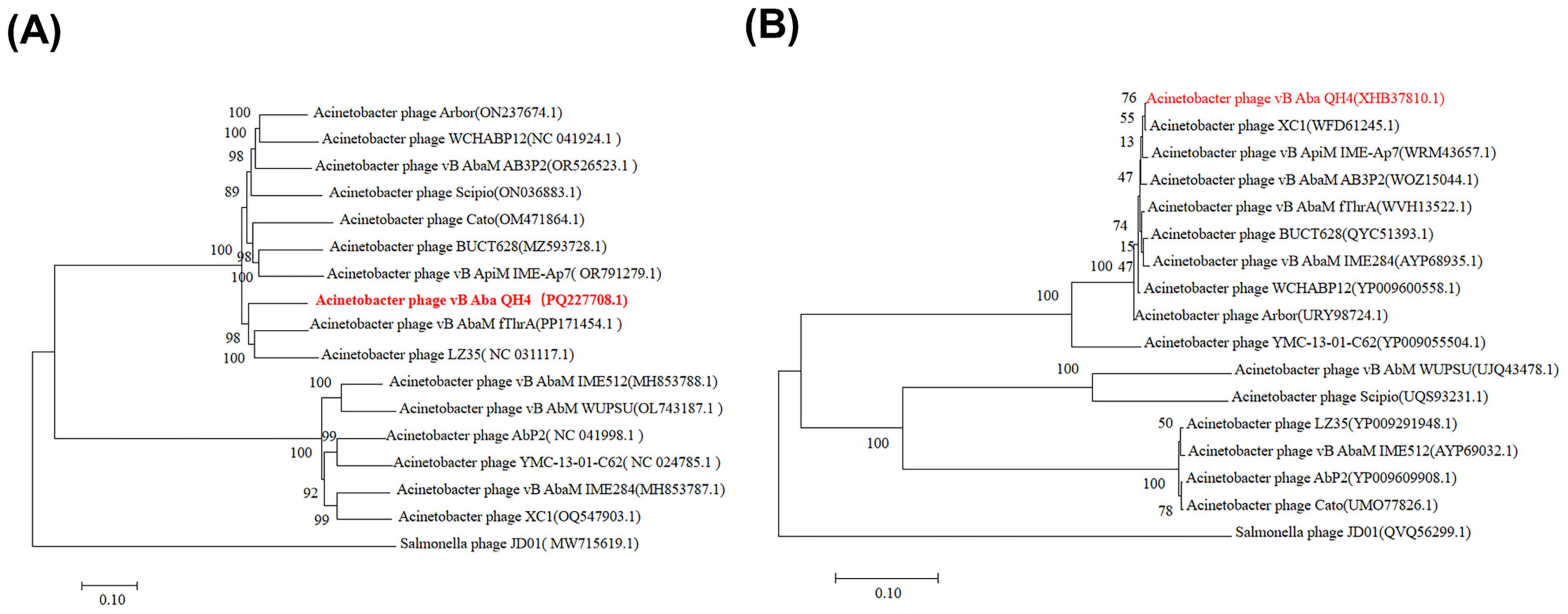
Phylogenetic trees of phage vB_Aba_QH4. Scale bar = 0.10 (represents the nucleotide/amino acid substitution rate per site). **(A)** Phylogenetic tree based on the complete genome sequence. **(B)** Phylogenetic tree based on the terminase large subunit protein sequence.

Based on the above content, the complete genome circle map was constructed using CGView (Figure 4). The complete genome of vB_Aba_QH4 was blasted using the BLASTn function of the NCBI online platform. The results showed that the coverage rate with the complete genome of XC1 (OQ547903.1) was 75%, and the Per. Ident was 95.37% (Supplementary Figure 2). Since the Per. Ident result depends on coverage, the average nucleotide identity (ANI) of the two complete genomes was analysed using the online tool VIRIDIC, resulting in 73% (Supplementary Figure 3). This is well below the 95% intra-species threshold recommended by ICTV, showing that vB_Aba_QH4 has been confirmed as a novel phage. The complete genome phylogenetic tree (Figure 5A) and the terminase large subunit protein phylogenetic tree (Figure 5B) were constructed using MEGA7 software. The complete genome phylogenetic tree indicates that vB_Aba_QH4 (PQ227708.1) is most closely related to Acinetobacter phage vB_AbaM_fThrA (PP171454.1) and Acinetobacter phage LZ35 (NC 031117.1). The phylogenetic tree of the large subunit of the terminase indicates that the Acinetobacter phage vB Aba QH4 (XHB37810.1) is most closely related to the Acinetobacter phage XC1 (WFD61245.1). According to the analysis by the PhageAI online platform, vB_Aba_QH4 has high integrity genome and “virulent” lifestyle (Supplementary Figure 4), indicating that vB_Aba_QH4 is a lytic phage. No antimicrobial resistance genes (AMRs) (Supplementary Figure 5) or bacterial virulence genes (Supplementary Figure 6) were detected in the Comprehensive Antibiotic Resistance Database and VirulenceFinder, proving the safety of vB_Aba_QH4 at the genetic level.

### 3.6 *In vitro* bacterial inhibition assay

**Figure 6:**
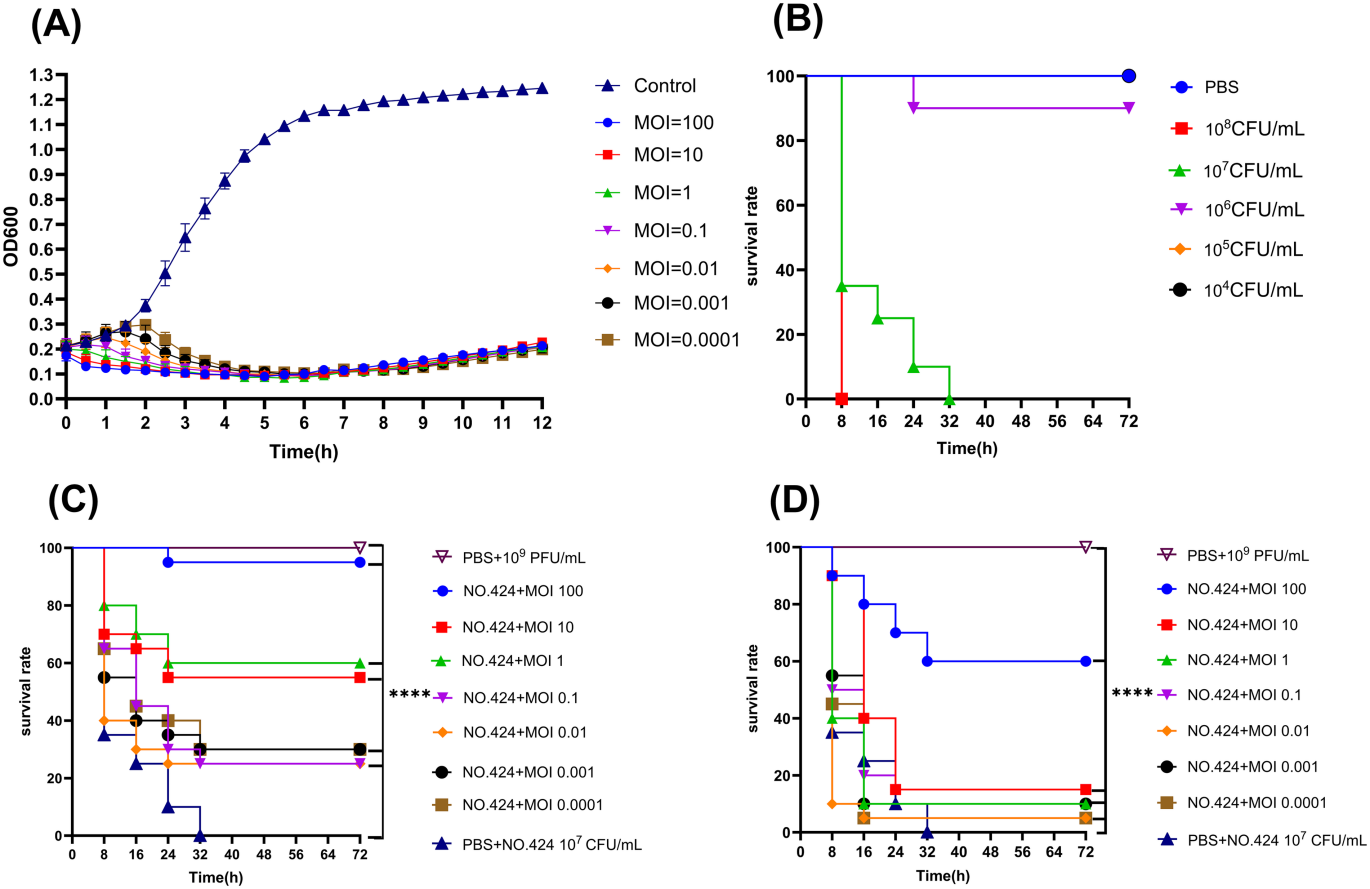
Antimicrobial effects of phage vB_Aba_QH4 *in Vitro* and *in vivo.* **(A)** Lytic activity of vB_Aba_QH4 against *A. baumannii* No.424 at different MOIs. **(B)** Survival curve of *G. mellonella* after infection with different *A. baumannii* No.424 concentrations. **(C)** Survival rate of *G. mellonella* during phage treatment. **(D)** Survival rate of *G. mellonella* after phage prophylaxis. ****Indicates statistical significance (*p* < 0.0001) by Mantel-Cox test.

In the *in vitro* bacterial inhibition test, the OD600 values of different MOI groups and positive control groups showed different changes (Figure 6A). The OD600 value of the control group maintained an increasing trend throughout the observation cycle with a final OD600 value of 1.245. During the first 6 h, When MOI = 100, 10, 1, the OD600 value continued to decline, from 0.186 to 0.095 on average, while MOIs of 0.1, 0.01, 0.001, and 0.0001 showed a biphasic change of firstly increasing and then decreasing, with MOI = 0.0001 having the highest peak value and the OD600 value being 0.297. *In vitro* inhibition of bacterial rate of the experimental groups all reached 90%. The *in vitro* inhibition rates of the experimental groups were all above 90%. After 6 h, the OD600 values of all groups with MOI ranging from 100 to 0.0001 showed an increase, but the increase was relatively small.

### 3.7 *In vivo* bacterial inhibition assay

The results of the *in vivo* inhibition experiments showed that the puncture group, the PBS control group, and the 10 μL of phage suspension at 10^9^ PFU/ml phage-treated group maintained 100% survival within 72 h, indicating that neither the experimental manipulation nor the phage itself had a significant effect on the survival of wax borer larvae. The survival rate of wax borer larvae at 10^5^ CFU/mL and 10^6^ CFU/mL bacterial concentrations was 100 and 90% within 72 h. The survival rate of wax borer larvae at 10^7^ CFU/mL bacterial concentration was 0% within 30 h. The survival rate of wax borer larvae at 10^8^ CFU/mL bacterial concentration was also 0% within 8 h. Based on the results of the experiments for the establishment of the infection model, it was determined that the optimal infection concentration was 10^7^ CFU/mL (Figure 6B). vB_Aba_QH4 larval survival was improved at all time points in both the treatment (Figure 6C) and prevention (Figure 6D) larval models. Survival rates at 72 h MOI 100–0.0001 were 95, 55, 60, 25, 25, 30, and 30%, respectively, in the treatment group. Survival rates of MOI 100–0.0001 after 72 h in the prevention group were 60, 15, 10, 15, 5, 10, and 5%, respectively.

## 4 Discussion

Our team successfully isolated and purified a novel virulent phage vB_Aba_QH4 (NCBI accession number: PQ227708) from hospital wastewater. The electron microscopic morphology, host profile, optimal number of infection replicates, one-step growth curve, adsorption curve, thermal stability, acid–base tolerance, UV sensitivity, chloroform assay, *in vitro* and *in vivo* bacteriostatic effects, complete genome sequencing, and functional analysis of vB_Aba_QH4 phage were determined.

The formation of uniformly translucent plaques on double agar medium using phage vB_Aba_QH4 is direct evidence showing that phage vB_Aba_QH4 is a lytic phage (Peng et al., 2020). The halo phenomenon implies that vB_Aba_QH4 has depolymerase activity (Kassa and Chhibber, 2011). And the PhageAI online platform predicts its lifestyle to be virulent, which also proves that it is a lytic phage.

Based the latest virus classification published by the International Committee on Classification of Viruses (ICTV) in 2024, phage vB_Aba_QH4 is classified in the genus Obolenskvirus, order Caudoviricetes. The narrow host range of vB_Aba_QH4 may limit clinical applications. On the one hand, *A. baumannii* frequently exhibits extensive serotype differentiation or genotypic variation. Single narrow-spectrum phages possess highly specific receptor recognition capabilities, thus typically lysing only specific sub-strains and proving inadequate against the highly heterogeneous pathogen communities encountered clinically. On the other hand, clinical infections frequently involve mixed infections with multiple pathogens (Khan et al., 2021). Narrow-spectrum phages are ineffective against non-target strains, failing to meet the therapeutic demands of complex infection scenarios. This fundamentally conflicts with the core clinical requirements of broad-spectrum coverage and rapid infection control. Currently, several research have been done to expand the host spectrum through the cocktail phage therapy (Harada et al., 2022), genome engineering modifications (Guo et al., 2021), and expression of lytic enzymes (Yang et al., 2014). The vB_Aba_QH4 genome predicted three proteins related to cleavage, and subsequently proposed to broaden the host spectrum using heterologous expression of cleavage enzymes.

The MOI of vB_Aba_QH4 was 0.001, which indicated that the phage concentration was 1,000 times lower than the bacterial concentration. The adsorption rate of vB_Aba_QH4 reached 99.9% at 6 min, which indicates its ability to complete adsorption rapidly. A latency period of 10 min and a lysis period lasting for 40 min suggest that it has an efficient replication and assembly ability, with a final burst of 460.5 PFU/cell. Prior to this, the latency period of vB_AbaS_qsb1 was 10 min, reaching a plateau phase at 100 min, with a burst size of 69 PFU/cell (Wang et al., 2025); the latency period of Abp9 was 30–40 min. The burst size is 158 PFU/cell (Jiang et al., 2020). vB_AbaS_TCUP2199 has an adsorption rate of 68.28% within 2 min, with a burst size of 196 PFU/cell (Mardiana et al., 2022). In comparison, vB_Aba_QH4 exhibits rapid adsorption capacity and high burst volume, providing a key theoretical basis for its rapid and effective diffusion within the host population.

A good understanding of the physicochemical stability factors of vB_Aba_QH4 facilitates phage storage, transport, and application. The phage maintains good biological activity at 4–50 °C while the range of the human body temperature is 35–42 °C, indicating that phage vB_Aba_QH4 can maintain good biological activity *in vivo*. It exhibits stable biological activity in the pH range of 3–11 and can tolerate the acidic and alkaline environments in most parts of the human body; therefore, it can be applied to the treatment of common clinical conditions caused by *A. baumannii*, such as respiratory pneumonia, bloodstream-borne infections, and infections of the lungs and the circulatory system. The chloroform test confirms that the coat of the phage does not contain a lipid envelope (Kusradze et al., 2016). When phage contain a lipid envelope, chloroform can dissolve the lipid components of the envelope, causing damage to the structure of the viral particles and loss of infectivity, as evidenced by a significant decrease in titre. It is agreed that chloroform does not affect the activity of non-enveloped phage, and the titre remains stable. vB_Aba_QH4 is sensitive to UV light, suggesting that it needs to be stored away from light to maintain its activity. The sensitivity to ultraviolet radiation indicates that the UV stability of phage vB_Aba_QH4 is markedly inferior to that of many conventional antibiotics or chemical drugs. The latter typically do not impose such stringent requirements on environmental light conditions.

Although shorter or longer incubations were not performed in this part of the study, it can be surmised based on the experimental results in this paper that longer incubation of vB_Aba_QH4 at temperatures higher than 60 °C and pH = 2 or 12 would result in gradual damage to vB_Aba_QH4 proteins or nucleic acids, which would exacerbate the drop in titer. The results of the chloroform experiments demonstrated that phage do not have a lipid envelope, so longer chloroform treatments should not affect vB_Aba_QH4 activity. Future studies could further validate this hypothesis and explore the specific mechanism of phage inactivation by shortening or lengthening the incubation time. This expected result is in line with the findings of Kusradze (Kusradze et al., 2016). In conclusion, vB_Aba_QH4 has good physical and chemical stability, which is a prerequisite for its future use in biopharmaceutical production or clinical applications.

Virulent phages inject their genes into host bacteria, use the host’s biosynthetic system to replicate their own genomes and synthesize proteins, and ultimately assemble complete viral particles, lysing the host bacteria and releasing offspring phages. vB_Aba_QH4 is one such virulent lytic phage. Multiple proteins are involved in or assist in the replication and transcription of vB_Aba_QH4. N-6-adenine-methyltransferase (ORF7) may be associated with catalyzing DNA N-6-adenine methylation resistant to degradation by host restriction endonucleases (Drozdz et al., 2011). Nucleotide kinase (ORF21) may regulate dNTP library balance and ensure replication continuity (Beauclair et al., 2020). DNA exonuclease (ORF85) and Erf-like (ORF86) annealing proteins play a role in DNA recombination or repair, preserving high-fidelity replication efficiency (Ahamed et al., 2019), while transcriptional regulator (ORF2) regulates the temporal expression of genes. Specifically, portal protein (ORF38) forms a pore or portal that facilitates DNA translocation (Cheepudom et al., 2019). Lysozyme proteins (ORF64, ORF79) are involved in host lysis, and the purified expression of lysozyme is becoming a promising option for phage preparations (Cremelie et al., 2024). Moreover, the head proteins (ORF34, ORF49, ORF50), tail proteins (ORF65, ORF66, ORF69, ORF72, ORF73), and head-tail junction protein (ORF57) collectively facilitate viral particle assembly, providing the structural foundation for host infection and self-replication. Furthermore, the terminase large subunit (ORF37), owing to its functional importance and sequence conservation, is commonly employed for constructing phylogenetic trees. Notably, vB_Aba_QH4 exhibits an ANI of less than 75% with all known phages in the NCBI reference database, indicating that it is a novel phage. Its complete genome and terminase large subunit protein phylogenetic trees intuitively illustrate vB_Aba_QH4’s phylogeny with other phages. In addition, its virulent lifestyle confirms that it is a lytic phage. The absence of antimicrobial resistance genes and bacterial virulence genes proves its safety.

In *in vitro* antimicrobial assays, an increase in OD600 values corresponds to an increase in bacterial counts, and vice versa. Within 6 h, the bacterial concentrations in the antimicrobial group showed a consistent downward trend for MOI = 100, 10, and 1. By analysing the extent and rate of reduction, it can be concluded that the higher the MOI, the more pronounced the lytic effect. This may be due to a large number of phages rapidly lysing bacteria, thereby inhibiting bacterial proliferation (Storelli et al., 2017). For MOI values of 0.1, 0.01, 0.001, and 0.0001, the bacterial concentration in the antimicrobial group first showed an upward trend before turning downward. The lower the phage concentration, the higher the peak and the later its appearance. This indicates that low-concentration phages require a certain amount of time for self-replication to achieve antibacterial effects. Notably, the peak at MOI = 0.0001 remains significantly lower than the control group. This experiment clearly demonstrates the phage’s ability to lyse bacteria. The OD600 value showed a slight upward trend after 6 h, which was temporarily attributed to the proliferation of phage-resistant bacteria (De Sordi et al., 2018). This phenomenon is consistent with the research conducted by Cheng Peng (Cheng et al., 2025). From the perspective of clinical application risks, the emergence of resistant strains within *in vitro* cultures over the short term indicates that phage vB_Aba_QH4 may face a rapid decline in efficacy when used as a monotherapy. The proliferative capacity of phage-selected resistant strains is independent of the initial MOI. Long-term interactions between phage and host bacteria can induce the emergence of resistant strains. It is worth noting that the emergence of phage-resistant bacteria is a key issue in clinical translation and application. Due to the diverse and complex mechanisms by which bacteria resist phage infection, in-depth exploration of the evolutionary patterns of resistance and its control strategies remains a key direction for future research. When 12 h, the OD600 value of the MOI groups remained significantly lower than that of the control group.

The *in vivo* antibacterial experimental animal model uses wax moth larvae because they are cost-effective, easy to handle, have innate immune responses similar to vertebrates, and do not raise ethical issues, making them an ideal model for studying immune responses (Insua et al., 2013). *In vivo* antibacterial experiment results indicate that the phage can significantly improve survival rates, with results similar to those of *in vitro* experiments. The vB_Aba_QH4 phage demonstrated excellent therapeutic efficacy in animal models. When MOI = 100, the survival rate of the treatment group reached 95%. Compared with other phages, the survival rate of the treatment group was 86.7% under MOI = 1 for phage (Lin et al., 2025); 90% under MOI = 50 for phage Abgy202162 (Tian et al., 2024a), and 80% under the same MOI conditions for phage Abgy202141 (Tian et al., 2024b). At MOI = 100, the survival rate of the vB_Aba_QH4 phage prevention group was 60%. This series of data fully proves that vB_Aba_QH4 has a good therapeutic effect. The higher the phage MOI, the more pronounced the improvement in survival rates. This may be because when MOI ≥ 1, a large number of phage particles simultaneously bind to bacterial surface receptors, directly disrupting cell membrane integrity through physical membrane perforation, leading to premature release of intracellular lysosomes (Lin et al., 2020). This process does not require completion of the replication cycle and can achieve rapid bactericidal effects within minutes. Additionally, within the infection site, a high MOI ensures increased spatial coverage of phage, enabling them to rapidly bind to their specific targets.

As observed in the experimental results, the preventive application of phage is less effective than their therapeutic application. This can perhaps be explained from three main aspects: first, during the preventive phase, the absence of host bacteria or extremely low bacterial loads prevents phage from achieving exponential amplification through the “proliferation-lysis” cycle, significantly reducing their antibacterial efficacy (Barrios-Hernández et al., 2019); second, in the absence of sufficient host bacteria, phages are easily cleared by the host immune system, making it difficult to maintain an effective therapeutic concentration (Chechushkov et al., 2021); more importantly, in the preventive state, the inflammatory response is weak, and vascular permeability is insufficient, severely limiting the phages’ directed penetration to the infection site. In contrast, the high bacterial load in therapeutic applications not only drives phage exponential proliferation but also significantly enhances phage tissue distribution through inflammation-mediated vascular dilation (Yu et al., 2019).

## 5 Conclusion

The existence of phages as antibacterial agents is gaining increasing attention from the scientific community. The biological properties of vB_Aba_QH4 showed that vB_Aba_QH4 has good physicochemical stability, efficient lysing activity, and good *in vitro* and *in vivo* bacterial inhibitory effects. Our genomic analysis also showed that vB_Aba_QH4 is a novel phage. vB_Aba_QH4 could be a potential antimicrobial agent in the prevention and treatment of *A. baumannii* infections; however, its narrow host spectrum may limit its wide application.

## Data Availability

The original contributions presented in the study are publicly available. This data can be found at: https://www.ncbi.nlm.nih.gov/nuccore/PQ227708. The names of the repositories and accession numbers can be found in the article and Supplementary material.
