# Supplementary figures and images for: Biological characterization and antibacterial effects of a novel virulent Acinetobacter baumannii phage strain vB_Aba_QH4 in vitro and in vivo
Source: Front Microbiol. 2025 Sep 26;16:1638702. doi: 10.3389/fmicb.2025.1638702 (PMC12513716; doi:10.3389/fmicb.2025.1638702)

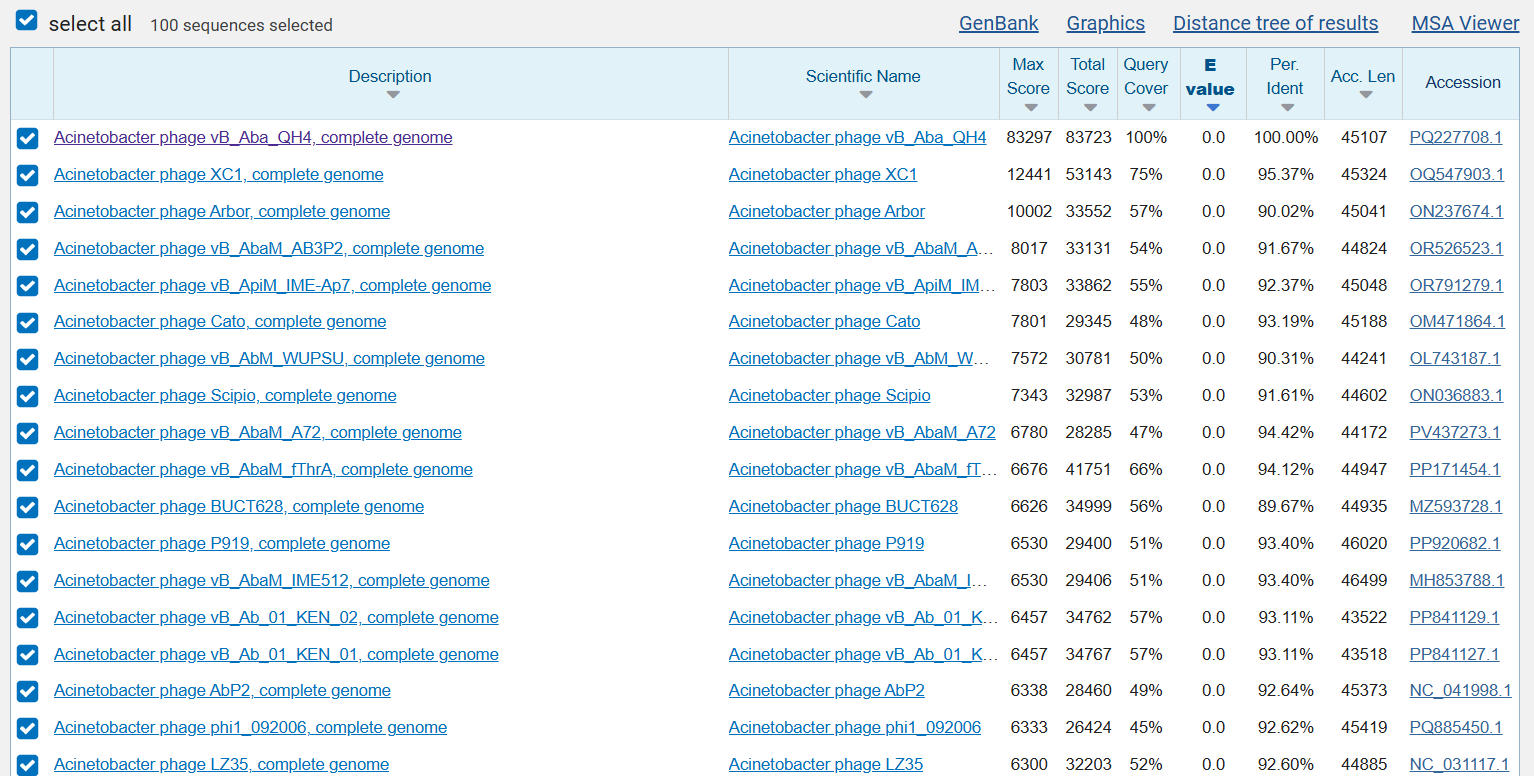

Supplement: Supplementary file 2 [file Image_2.TIF]

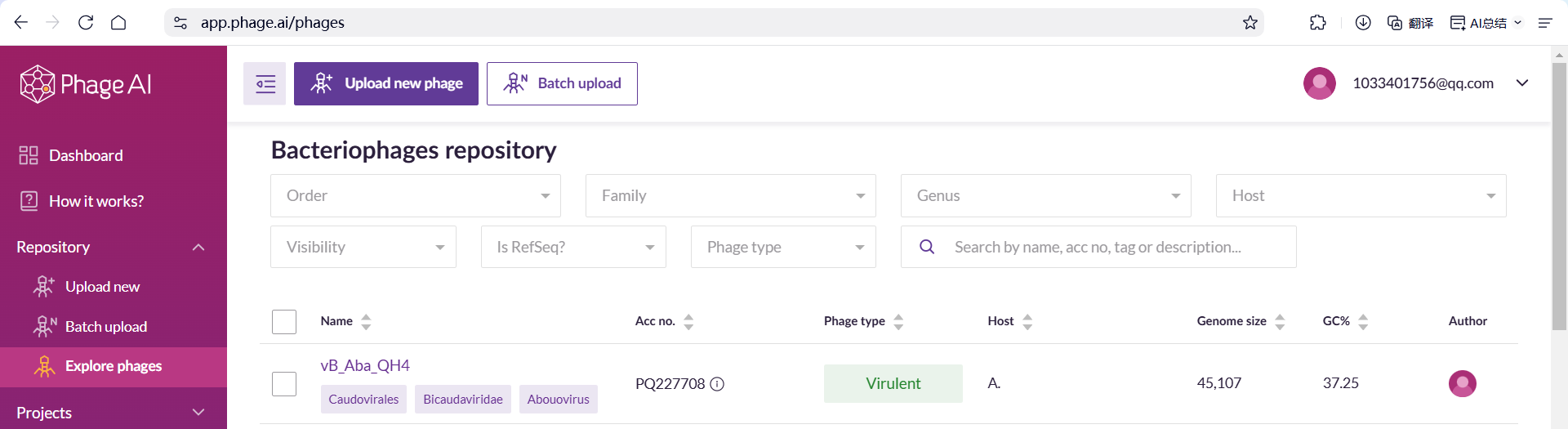

Supplement: Supplementary file 4 [file Image_4.TIF]

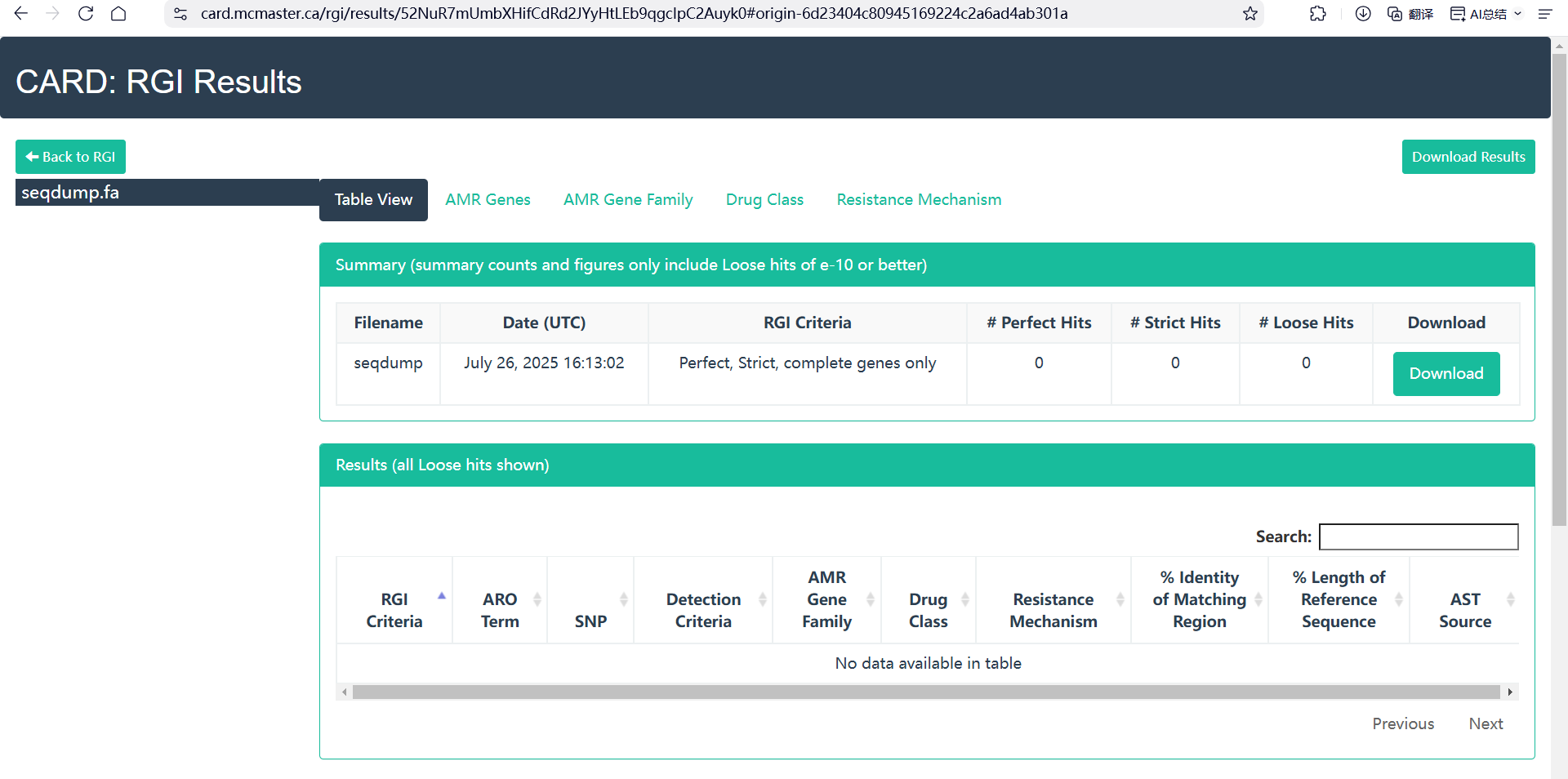

Supplement: Supplementary file 5 [file Image_5.TIF]

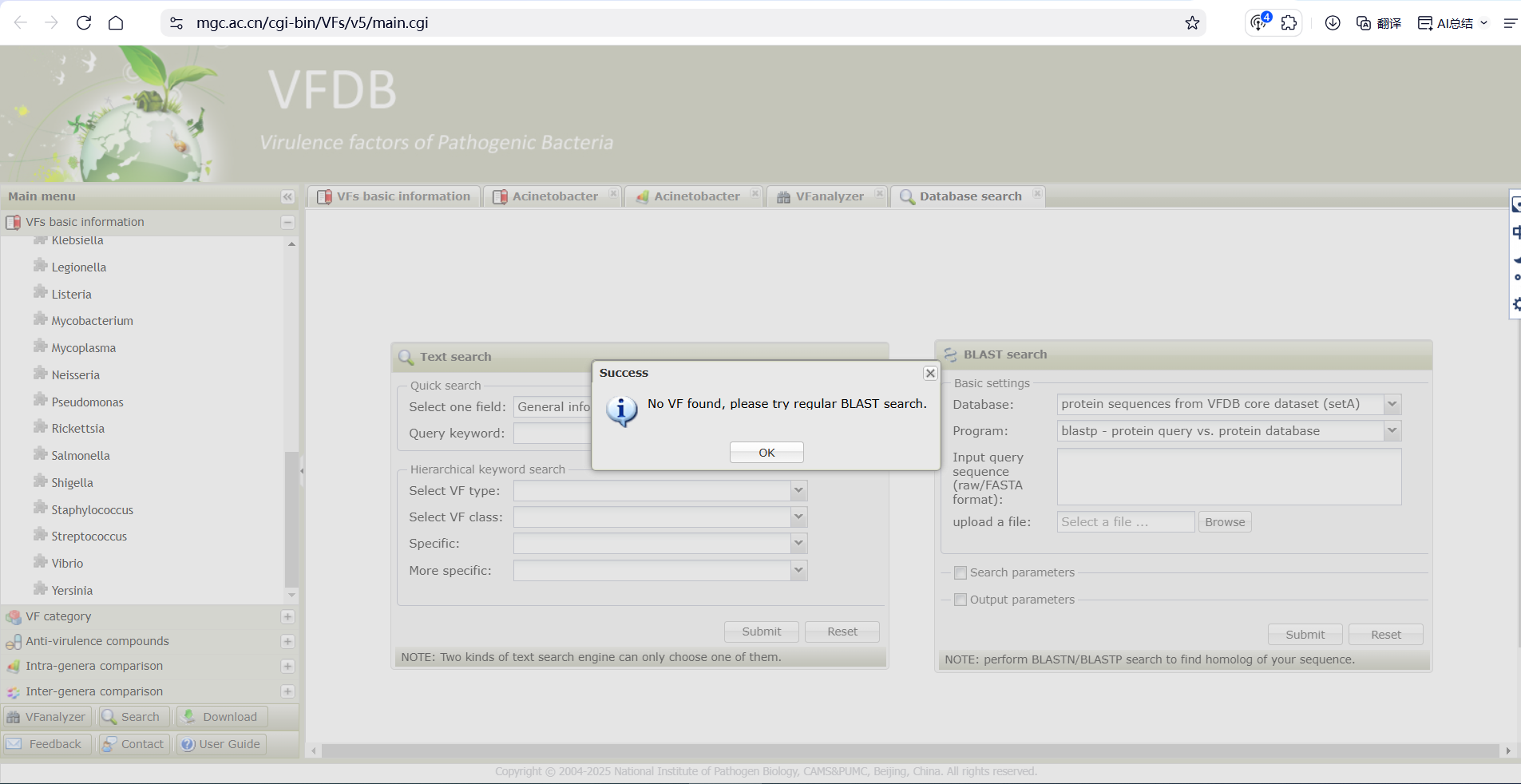

Supplement: Supplementary file 6 [file Image_6.TIF]
